# Chitosan as an Antimicrobial, Anti-Insect, and Growth-Promoting Agent for Potato (*Solanum tuberosum* L.) Plants

**DOI:** 10.3390/molecules29143313

**Published:** 2024-07-13

**Authors:** Aleksandra Steglińska, Adriana Nowak, Regina Janas, Mieczysław Grzesik, Krzysztof Śmigielski, Dorota Kręgiel, Beata Gutarowska

**Affiliations:** 1 Department of Environmental Biotechnology, Lodz University of Technology, Wólczańska 171/173, 90-530 Łódź, Poland; adriana.nowak@p.lodz.pl (A.N.); krzysztof.smigielski@p.lodz.pl (K.Ś.); dorota.kregiel@p.lodz.pl (D.K.); 2 The National Institute of Horticultural Research, Konstytucji 3 Maja 1/3, 96-100 Skierniewice, Poland; regina.janas@inhort.pl (R.J.); mieczyslaw.grzesik@inhort.pl (M.G.)

**Keywords:** potato, chitosan, biological control, cytotoxicity

## Abstract

A growing trend in plant protection is replacing chemical preparations with environmentally friendly biological compositions. Chitosan, due to its biocompatibility, biodegradability, and bioactivity, is an effective agent against plant diseases. The purpose of the study was to evaluate chitosan as a potential biopesticide for potato plants. Three variants of chitosan were tested: high (310–375 kDa, >75% deacetylated), medium (190–310 kDa, 75–85% deacetylated), and low (50–190 kDa, 75–85% deacetylated) molecular weight. The chitosan variants were dissolved in lactic and succinic acids and tested for antibacterial and antifungal properties against eight strains of mould and two strains of bacteria responsible for potato diseases. The possible cytotoxicity of chitosan was evaluated against different cell lines: insect Sf-9, human keratinocyte HaCaT, and human colon carcinoma Caco-2. The bioprotective activities of the chitosan were also evaluated in situ on potato tubers. Chitosan inhibited the growth of almost all the selected phytopathogens. The most active was medium molecular chitosan in lactic acid. This formula was characterized by low toxicity towards human cells and high toxicity towards Sf-9 cells. It was also found to have positive effects on the growth of stems and roots, gas exchange, and chlorophyll index in potato plants. Selected chitosan formulation was proposed as a functional biopesticide for potato protection against phytopathogens.

## 1. Introduction

The potato (*Solanum tuberosum* L.) is the fourth most cultivated crop in the world, with a production of 376 million tonnes in 2021, after corn (1.15 billion tons), wheat (780 million tons), and rice (510 million tons) [1]. European potato production has been falling, halving in the period 1961–2019. Nonetheless, the increasing demand from 2001 to 2019 for processed potato products, notably French fries and chips, along with the development of processing techniques for dried potatoes and starch, suggest this trend may reverse in the coming years [2]. Potatoes, like other crops, are exposed to various phytopathogens (bacteria, fungi, viruses, and insects) that reduce both their quality and yield, with impacts for food security globally. It is estimated that 10–16% of the world’s crops are lost each year due to damage caused by phytopathogens, representing a financial loss of $220 billion [3]. Global losses of potatoes due to phytopathogens are estimated to be even higher, averaging 17.2% [4].

The most important bacteria causing potato tuber diseases are *Pectobacterium carotovorum* (soft rot disease), *Clavibacter michiganensis* subsp. *Sepedonicus* (ring rot), *Ralstonia solanacearum* (bacterial wilt), and *Streptomyces scabiei* (common scab). These pathogens cause severe damage to both tubers and crops, including soft rot (*Pectobacterium carotovorum*), commonly known as bacterial wilt, black leg (often associated with *Pectobacterium* species), ring rot (*Clavibacter michiganensis* subsp. *Sepedonicus*), pink eye (a condition associated with various bacterial and environmental factors), and common scab (*Streptomyces scabiei*) [5]. Several genera/species of moulds can also infect potatoes, causing various symptoms in tubers. Key fungal pathogens leading to notable diseases in potatoes include *Fusarium* sp. (causing sunken brown necrotic areas known as dry rot), *Alternaria* sp. (responsible for alternariosis), *Colletotrichum coccodes* (causing potato black dot), *Phoma exigua* (leading to gangrene), *Rhizoctonia solani* (associated with black scurf), *Helminthosporium solani* (causing silver scurf), and the fungus-like oomycete *Phytophthora infestans* (responsible for late blight) [6,7,8,9,10]. In addition, a large number of insect pests infest potato crops. These insects either damage the tubers directly or transmit devastating pathogens while feeding on leaves/stems. Notable pests include the fall armyworm (*Spodoptera frugiperda*), tuber moths (*Phthorimaea operculella*), cutworms (*Agrotis ipsilon*), Colorado potato beetles (*Leptinotarsa decemlineata*), and potato aphids (*Macrosiphum euphorbiae*) [11]. 

The primary strategy for managing fungal and bacterial diseases in crops is the application of synthetic pesticides. Although these chemicals are effective in controlling pathogens, they pose significant risks to both human health and the environment. The toxicity of chemical pesticides is variable and depends on many factors, such as the dose, degradation time, rate of penetration, and weather conditions during application. The toxic effects of chemical pesticides on human and animal health are linked to their impact on the nervous system, enzyme activity, skin and mucous membranes, metabolism, and the levels of free oxygen radicals [12]. According to a 2020 report commissioned by the EU, pesticide residues were detected in 26.2% of potato samples, and the exposure limit was exceeded in 0.8% of samples. More than one pesticide residue was found in 4.2% of the samples [13].

There is a pressing need to find effective and environmentally friendly methods to protect against potato phytopathogens. Biopesticides offer a promising solution. The EPA (United States Environmental Protection Agency) defines biopesticides as pesticides derived from natural materials [14]. They include biochemical pesticides, microbial pesticides, and plant-incorporated protectants. Currently, biopesticides account for only about 5% of the global pesticide market. However, they are expected to surpass synthetic pesticides in usage by 2050 [15]. Microbial pesticides can contain bacteria, fungi, viruses, or protozoans as their active ingredients. Previous research on the microbial sources of biopesticides has highlighted the use of bacteria: *Pseudomonas fluorescens*, *Bacillus subtilis* [16], *B. velezensis* [17], *Lactiplantibacillus plantarum* [18], *B. thuringiensis* [11]; fungi *Epicoccum nigrum* [19], *Metschnikowia pulcherrima* [20,21,22], *Trichoderma* sp. [23], *Aureobasidium pullulans* [24], *Beauveria bassiana* [25], or *Paecilomyces farinosus* [26]. Biochemical pesticides contain substances that interfere with insect mating, such as insect sex pheromones, as well as various scented plant extracts that attract insect pests to traps or have antimicrobial potential. The best known plant extracts for combatting plant diseases include garlic (*Allium sativum* L.), thyme (*Thymus vulgaris* L.), caraway (*Carum carvi* L.) [27], pomegranate (*Punica granatum* L.) [28], eucalyptus (*Eucalyptus globulus* Labill), orange peel (*Citrus aurantium var. sinensis* L.), and cistus (*Cistus ladanifer* L.) [29]. A recent study by the authors of the present study on garlic extract conducted in situ showed an especially high protective effect against microbial diseases during the growth and storage of potatoes [27].

Over the last decade, chitosan has gained increasing popularity as a biopesticide due to its high antimicrobial and antifungal activities [30]. Chitosan has found applications in a wide range of industries, including textiles, cosmetics, beverage processing, packaging, and water treatment [31]. Chitosan is a natural polymer, originating mostly from crabs and shrimps. It is composed of β-1,4-D-glucosamine and N-acetyl-D-glucosamine monomers [32]. The activity of chitosan depends on its molecular weight and degree of acetylation, as well as the pH of the media and the concentration of chitosan [30]. Its mode of antimicrobial action is still not fully understood. The most widely accepted hypothesis is that chitosan interacts with negatively charged components on the microbial cell surface. These interactions are facilitated by the polycationic structure of chitosan, which leads to the leakage of intracellular organelles that cause cell death [33]. Due to its antimicrobial properties, chitosan is considered an effective coating for biocontrol in postharvest products [34]. In 2022, the U.S. Environmental Protection Agency (EPA) recognized chitosan’s low-risk profile by adding it to the list of active ingredients eligible for the minimum risk pesticide exemption. The Agency’s analysis of the available data concluded that chitosan and its salts have low toxicity towards humans and pose no environmental risk [31].

There has been limited research on utilizing chitosan for protecting potato plants. An intriguing approach involved the application of *Streptomyces* sp. spores encapsulated in chitosan beads as a novel biocontrol method against the potato common scab [35]. The antifungal activity of chitosan against *A. tenuissima* and its ability to induce defence responses in potato tubers have been studied previously [36]. However, there remains a lack of extensive research specifically addressing the use of chitosan on stored potato tubers, including assessments of its antimicrobial activity against a broad spectrum of pathogens. The aim of the study was to evaluate chitosan as a potential biopesticide for potato tubers. Here, we provide an original and comprehensive evaluation of chitosan as a natural biocontrol agent for potato protection. We assessed the effectiveness of using chitosan as a bioprotective agent against ten potato phytopathogens. To address the environmental aspects of using chitosan as a biocontrol agent, we evaluated chitosan cytotoxicity against three different cell lines: insect, human skin, and carcinoma colonies. We tested variants of chitosan with different molecular weights (high (310–375 kDa, >75% deacetylated), medium (190–310 kDa, 75–85% deacetylated), and low (50–190 kDa, 75–85% deacetylated)) in solutions with lactic or succinic acid. The most active formulation in terms of antibacterial and antifungal properties was selected for further studies. The possible cytotoxicity of the chitosan formulation was evaluated against the cell lines Sf-9 (*Spodoptera frugiperda*), HaCaT, and Caco-2. We also determined the bioprotective activities of the selected chitosan on potato tubers in situ during storage. Finally, we evaluated the influence of the chitosan formulation on the growth and physiological activity of the potato plants in a greenhouse experiment.

## 2. Results and Discussion

### 2.1. In Vitro Evaluation of Antimicrobial Activities of Chitosan Solutions against Potato Pathogens

Since the first description of chitosan by Henri Braconnot in 1811, chitosan has been studied extensively in terms of its structure and antimicrobial activities in vitro [30,33,37]. This biopolymer has several interesting physicochemical properties. It is possible to dissolve chitosan under acidic conditions, and it can adopt several conformations or structures that can be used to modulate its superficial composition for specific applications [38]. Over the last decade, the number of studies on the use of chitosan as a postharvest plant protection agent has increased linearly [34]. However, there are only a few studies in the literature concerning chitosan’s potential use as a biocontrol agent for potatoes [35]. The main aim of the present study was to provide an extensive report on the antibacterial and antifungal properties of chitosan against seed potato phytopathogens, evaluated in vitro. The antimicrobial activities of 6 chitosan formulations were assessed against 10 potato pathogens. The results are presented in Table 1.

All the chitosan variants dissolved in lactic acid inhibited the growth of at least 8 of the 10 tested phytopathogens. Those dissolved in succinic acid inhibited at most seven tested species. Interestingly, no antimicrobial activity against the mould *C. coccodes* was observed. No previous studies have assessed the antimicrobial activity of chitosan specifically against *C. coccodes*. However, several have confirmed its effectiveness against other species that can also cause anthracnose in mango fruits [39]. Commercially available low molecular weight chitosan in acetic acid and sodium acetate solution (0.1% *w*/*v*) has been reported to show antifungal activity against *Colletotrichum* species (*C. chrysophilum*, *C. siamense*, and *C. musae*). A similar effect was observed by Ramos Berger et al. [40] for chitosan (500 ppm solution in acetic acid) against *C. siamense*, *C. asianum*, *C. fructicola*, *C. karstii*, and *C. tropicale*.

In our study, high molecular weight chitosan proved the least effective at inhibiting the tested pathogens. The highest MIC values were recorded for *S. scabiei* (0.8333–3.333 mg/mL) and *A. alternata* (0.4167–3.333 mg/mL or no MIC for half of the variants). Fungi species *F. oxysporum* (MIC: 0.05208–0.1041 mg/mL, MBC/MFC: 0.2083–0.4167 mg/mL), *F. sambucinum* (MIC: 0.05208–0.4167 mg/mL, MBC/MFC: 0.2083–1.666 mg/mL), *P. exigua* (MIC: 0.05208–0.2083 mg/mL, MBC/MFC: 0.1041–0.4167 mg/mL), and *A. solani* (MIC: 0.05208–0.2083 mg/mL, MBC/MFC: 0.4165–1.666 mg/mL) were the most susceptible to the chitosan solutions. For the MM-L variant, the susceptibility series was as follows: *P. exigua* (MIC: 0.1041 mg/mL) > *F. oxysporum* (MIC: 0.1041 mg/mL) > *A. solani* (MIC: 0.1041 mg/mL) > *A. tenuissima* (MIC: 0.1041 mg/mL) > *P. carotovorum* (MIC: 0.2083 mg/mL) > *F. sambucinum* (MIC: 0.4167 mg/mL) > *A. alternata* (MIC: 0.4167 mg/mL) > *S. scabiei* (MIC: 0.8333 mg/mL) > *R. solani* (MIC: 1.666 mg/mL) > *C. Colletotrichum* (MIC: not detected). Our results for the antifungal activity of chitosan against *R. solani* are in agreement with a study by Liu et al. [41], in which different types of chitosan had only an inhibitory rather than fungicidal effect. In our research, only one of the six types of chitosan (MM-L) had a fungicidal effect on *R. solani*. Ren et al. [42] also found that chitosan can effectively inhibit the growth of *F. oxysporum*.

Other studies on the antimicrobial properties of chitosan against microorganisms that might infest potatoes have mentioned the synergistic effects of chitosan with tetramycin (*A. tenuissima*) [43], sodium silicate (*A. alternata*) [44], and thyme essential oil (*P. carotovorum*) [45], as well as the enhanced antimicrobial effects of chitosan subjected to γ-irradiation (*Alternaria* sp., *Fusarium* sp.) [46]. Chitosan alone and unirradiated can also be an effective antimicrobial agent, but to a lesser degree. Another approach involves using chitosan combined with microorganisms. Lin et al. [47] successfully used chitosan and lactic acid bacteria to control *P. carotovorum* and two more phytopathogens. El-Morsy et al. [48] developed a method that combines chitosan nanoparticles with *Trichoderma longibrachiatum* and *Penicillium polonicum* to inhibit the growth of *Fusarium equiseti*. To the best of our knowledge, this is the first study to report the antimicrobial activity of a chitosan acid solution against *P. exigua*.

The MM-L and LM-L variants of the chitosan solutions demonstrated the broadest spectra of activity, inhibiting growth in 9 of the 10 tested phytopathogens. However, the concentrations of chitosan solution necessary to inhibit the growth of phytopathogens were lower in the case of MM-L (medium molecular weight chitosan dissolved in lactic acid). Therefore, as the agent with the greatest bioprotective potential, this variant was selected for use in further studies. 

### 2.2. Cytotoxicity of MM-L Chitosan Solution against Selected Cell Lines

We studied the cytotoxicity of the MM-L chitosan formulation with the highest antimicrobial properties against selected cell lines: Sf-9 (from the ovaries of *S. frugiperda* larvae), HaCaT (from human keratinocyte cells), and Caco-2 (from human colon adenocarcinoma cells). These lines were selected to test the environmental toxicity of the chitosan formulation towards both human and animal cells. Sf-9 cells were chosen because they are the best-established insect cells, being widely used in agricultural toxicity studies. Keratinocytes constitute almost 95% of the epidermis, and are usually the first to come into contact with any extraneous chemical compounds. Caco-2 cells are an in vitro model of the intestinal barrier. This cell line is the most widely used research model for studying the interaction of gastrointestinal cells with various chemical compounds. Chitosan residues can also be swallowed and reach the gastrointestinal tract.

**Figure 1:**
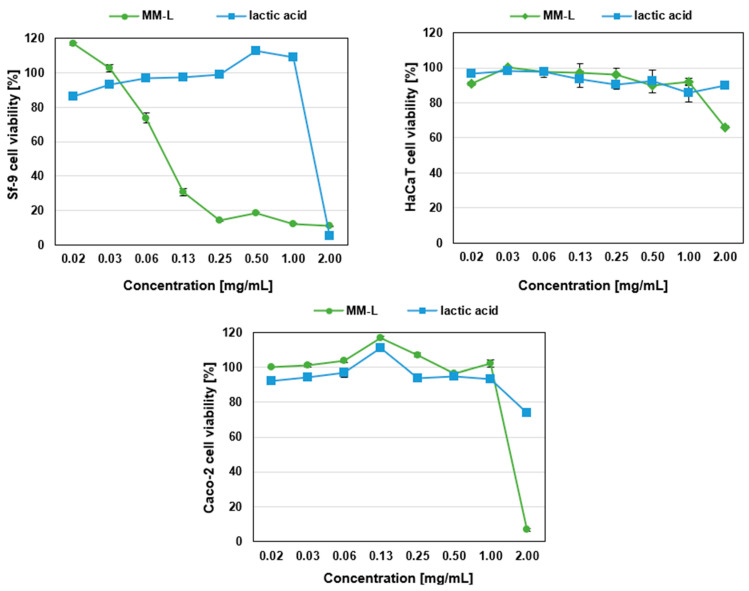
Cytotoxicity of MM-L chitosan solution against Sf-9, HaCaT, and Caco-2 cell lines. MM-L—medium molecular weight chitosan dissolved in lactic acid.

The results of cytotoxicity tests against the selected cell lines are presented in Figure 1.

The selected MM-L chitosan formulation showed a strong cytotoxic effect against the Sf-9 insect cell line. Sf-9 cell viability decreased with increasing chitosan concentrations (0.016–2 mg/mL). This can be explained by the cytotoxicity of MM-L, especially since the cytotoxicity of the solvent, lactic acid, was low. However, the low cell viability at the highest concentration of 2 mg/mL may be partly related to the activity of the lactic acid used to dissolve the chitosan. The MM-L chitosan solution (0.016–1 mg/mL) showed a weak cytotoxic effect against human epidermal cells from the HaCaT line, with cell viability in the range of 90.9–100.1%. A rapid increase in cytotoxicity was, however, observed at a concentration of 2 mg/mL, when the cell viability decreased by up to 66.1%. In the case of Caco-2 cancer cells, very high cytotoxicity was recorded for a concentration of 2 mg/mL, with cell viability decreasing to 6.6%. This high level of cytotoxicity may be attributed to the combined effects of chitosan and lactic acid. Significantly, all cell lines were sensitive to the highest MM-L concentration tested (2 mg/mL). MM-L, in combination with lactic acid at the highest concentration, induced the strongest cytotoxicity against all three cell lines. This may be due to the induction of oxidative stress in cells through the generation of reactive oxygen species by chitosan [49]. The exact mechanisms explaining the cytotoxicity of chitosan in different cell lines (human, insect, normal, and tumour) are poorly understood. In summary, the chitosan formulation used in our study has a slight toxic effect on human cells and may have a toxic effect on insects. Our results align with the literature data. According to the literature, chitosan is innate, biocompatible, and non-toxic to living cells and tissue. Chitosan biocompatibility has been tested in vitro using different types of cells, including fibroblasts, keratinocytes, and hepatocytes, as well as myocardial and endothelial cells. All results showed no toxicity [50]. However, Wiegand et al. [51] reported that chitosan exhibited a cytotoxic effect on the HaCaT cell line, with the extent of cytotoxicity varying based on molecular weight, duration of exposure, and concentration. Specifically, concentrations of 0.5% and 1% of 120 kDa chitosan were found to be cytotoxic after just a 2 h incubation period, whereas chitosan oligosaccharide required at least 24 h of incubation to exhibit a similar effect.

Another study conducted by Kim et al. [52] showed that fully deacetylated quaternized chitosan was nearly non-cytotoxic towards the HaCaT cell line at a concentration of 50 µg/mL. Several studies have confirmed that chitosan and quaternized chitosan have little to no negative impact on the viability of Caco-2 cells [53,54]. Chitosan nanoparticles were found to be cytotoxic to Caco-2 cells only at a lower pH of 6 [55]. Therefore, traces of chitosan in the environment or even in food do not have a significant impact on human health. As a non-toxic compound, chitosan can be processed in a broad range of sustainable, commercial applications [56]. A recent study showed that a chitosan preparation was effective against common house mosquitos, *Culex pipiens* [57]. Our results suggest that chitosan could be a promising tool for the eco-friendly control of various insect species that that are (i) pathogens and (ii) important vectors of diseases. 

### 2.3. In Situ Evaluation of Antimicrobial Activities of MM-L Chitosan Solution against Potato Phytopathogens

Previous researchers have investigated the use of chitosan to control plant diseases during storage. Limon et al. [58] used an acetic chitosan solution (20 g/L) as a coating on mango. The coating reduced the severity of anthracnose in mango by 5–6 times. Chitosan has also been shown to be effective at controlling rhizome rot caused by *F. oxysporum* in ginger during storage [59], as well as winter jujube rot caused by *A. alternaria* [44]. In these studies, chitosan not only inhibited the development of disease but also prevented water loss and discoloration. In our research, we conducted an in situ experiment to assess the ability of MM-L chitosan to control phytopathogens on seed potatoes during storage. The results are presented in Table 2. 

The results of in situ experiments correlated with the first, in vitro stage of the research. The MM-L chitosan solution inhibited the development of selected fungi of the genera *Fusarium*, *Alternaria*, *Phoma*, and *Rhizoctonia*, as well as selected bacteria of the genera *Pectobacterium* and *Streptomyces*. These findings are in agreement with [36], in which chitosan was found to have direct antifungal properties against *A. tenuissima* on stored potatoes. The literature describes chitosan as having bacteriostatic and bactericidal effects, often without making any distinction between these two terms. More recent studies tend to focus more on the bacteriostatic effect of chitosan, although the exact mechanism of action against pathogens is not well known. The literature identifies several generally accepted mechanisms by which chitosan exerts its antimicrobial effects against microorganisms: disrupting the cell membrane/cell wall; interacting with microbial DNA; chelation of nutrients; and formation of a dense polymer film on the surface of bacterial cells [50]. 

The most sensitive phytopathogens to chitosan were fungi from the species *F. oxysporum* (100% inhibition), *R. solani* (80%), and *P. exigua* (80%). No inhibition of phytopathogen growth was observed on seed potatoes inoculated with *C. coccodes*. Sun et al. [60] reported a reduction in the severity of dry rot in potato tubers dipped in 0.25% chitosan dissolved in 0.5% lactic acid. Several studies have demonstrated that chitosan effectively controls diseases caused by *P. infestans*, whether applied to potato tuber slices, potted potato plants, or field-grown potatoes [61,62]. Chitosan nanoparticles were also able to reduce the incidence and severity of disease caused by *R. solanacearum* on potato plants [63]. The mechanisms by which chitosan inhibits fungal growth are not yet fully understood. However, a theory outlining several key steps has been proposed: (i) Chitosan as a cationic polymer interacts with the negatively charged cell surface and destabilizes the electrical charge of cell wall. (ii) When the negative charge is decreased, the concentration of the cations at the surface is also affected. The difference between internal and external concentration leads to the efflux of cations. (iii) The efflux of cations causes hyperpolarization of the plasma membrane, causing an increased uptake of Ca^2+^ and the loss of negatively charged molecules (nucleotides, phosphates, and substrates for enzyme reaction), affecting cell metabolism and altering important metabolic pathways [50].

### 2.4. Greenhouse Experiments

**Figure 2:**
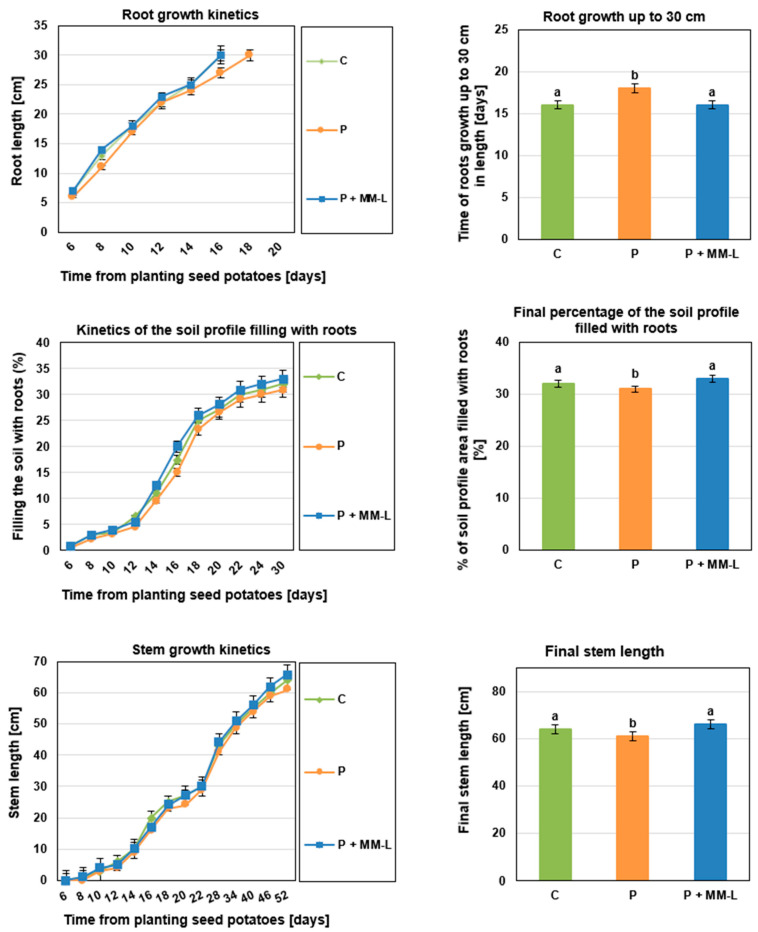
Root and stem growth of “Impresja” seed potato plants treated with MM-L chitosan solution and inoculated with phytopathogens. C—control potato, not treated with chitosan solution and not inoculated with pathogens; P—potato sample inoculated with pathogens only; P + MM-L—potato sample treated with MM-L chitosan solution and inoculated with pathogens. Different letters above the bars indicate a significant (*p* < 0.05) difference (one-way ANOVA with Duncan’s multiple range test).

**Figure 3:**
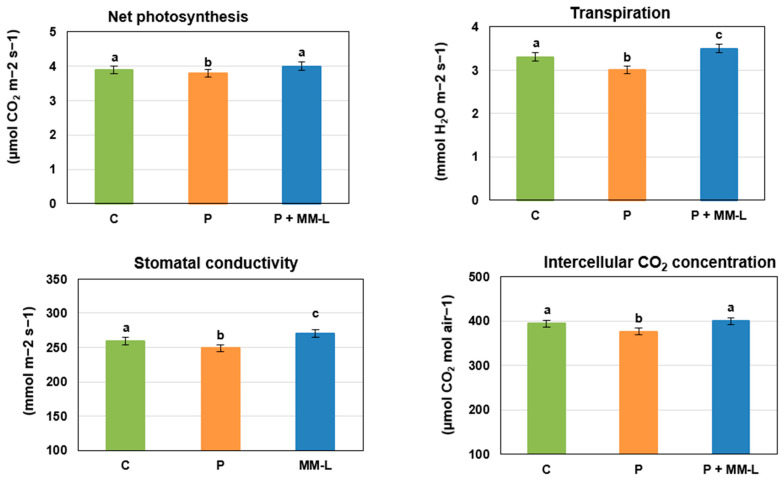
Gas exchange in leaves obtained from “Impresja” seed potatoes treated with MM-L chitosan solution and inoculated with phytopathogens. C—control potato, not treated with chitosan solution and not inoculated with pathogens; P—potato sample only inoculated with pathogens; P + MM-L—potato sample treated with MM-L chitosan solution and inoculated with pathogens. Different letters above the bars indicate a significant (*p* < 0.05) difference (one-way ANOVA with Duncan’s multiple range test).

**Figure 4:**
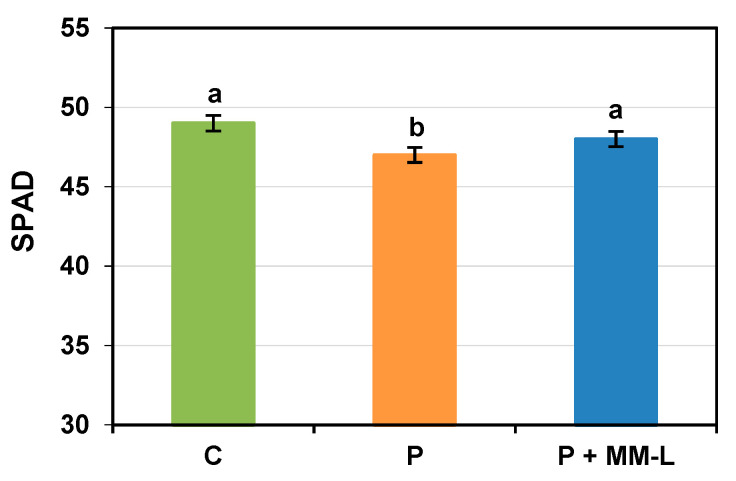
Index of chlorophyll content in leaves obtained from “Impresja” seed potatoes treated with MM-L chitosan solution and inoculated with phytopathogens. C—control potato, not treated with chitosan solution and not inoculated with pathogens; P—potato sample only inoculated with pathogens; P + MM-L—potato sample treated with MM-L chitosan solution and inoculated with pathogens. Different letters above the bars indicate a significant (*p* < 0.05) difference (one-way ANOVA with Duncan’s multiple range test).

Chitosan is used in agriculture not only to control plant pathogens but also as a growth promotion factor for fruits, vegetables, and flowers such as tomatoes, oregano, pepper, orchid, freesia, rape, wheat, strawberries, and lettuce [64,65,66]. In the final stage of our research, we investigated the impact of the selected MM-L chitosan solution on the germination, development, and physiological activity of seed potatoes. The seed potatoes were grown in microcosms in a greenhouse experiment. The results are presented in Figure 2, Figure 3 and Figure 4. 

Inoculation with a mixture of pathogens and application of the MM-L chitosan solution did not affect the ability of the seed potatoes to germinate (100% germinated). The MM-L solution showed positive effects on the growth of stems and roots, gas exchange, and the chlorophyll content index (Figure 2, Figure 3 and Figure 4). Potatoes treated with MM-L and infected with pathogens achieved a higher final stem length (66 cm) than potatoes only inoculated with pathogens (61 cm) and control potatoes (64 cm) (Figure 4). This indicates that chitosan has growth promoting activities. The values for root growth, and percentage of the soil profile filled with roots, chlorophyll content index, and gas exchange were comparable to the results for control potatoes, not infected with pathogens. Therefore, the application of MM-L eliminated the negative impact of phytopathogen diseases on these growth indicators. These results are consistent with a study by Muley et al. [46,67], where potato plants treated with chitosan or γ-irradiated chitosan foliar spray were characterized by improved shoot length and increased chlorophyll content. An increase in the activity of defence enzymes (superoxide dismutase, peroxidase, and catalase) was also observed. Other previous works have indicated that soluble chitosan, sprayed in greenhouse experiments on potato minitubers, increased tuber yield, water loss reduction [68], shoot fresh weight, and the number of tubers [69]. However, the plant growth promoting activities depended on the concentration of chitosan used as well as the cultivation season. The concentration of chitosan solution should therefore be selected carefully for each agricultural application. The plant growth promoting activities of chitosan are mostly considered to be the result of intensified nitrogen and nutrient uptake, increased water use efficiency, and the positive impact on mycorrhization [66].

## 3. Materials and Methods

### 3.1. Biological Materials Used in the Research

#### 3.1.1. Chitosan Solutions

Six variants of chitosan formulations were used: three types of commercial chitosan with low (50–190 kDa, 75–85% deacetylated), medium (190–310 kDa, 75–85% deacetylated), and high (310–375 kDa, >75% deacetylated) molecular weight (Sigma-Aldrich, Saint Louis, MO, USA) in two types of organic acid, lactic (Akwawit, Leszno, Poland) and succinic (Warchem, Warsaw, Poland). Chitosan is insoluble in water but soluble in dilute aqueous acidic solution. Lactic and succinic acids were selected because of their biodegradability and potential antimicrobial activity. The variants of chitosan solutions were as follows: HM-L—high molecular weight chitosan dissolved in lactic acid; MM-L—medium molecular weight chitosan dissolved in lactic acid; LM-L—low molecular weight chitosan dissolved in lactic acid; HM-S—high molecular weight chitosan dissolved in succinic acid; MM-S—medium molecular weight chitosan dissolved in succinic acid; LM-S—low molecular weight chitosan dissolved in succinic acid. Stock solutions of chitosan in 10 mg/mL acid were obtained by magnetic stirring (400 rpm, IKA^®^ C-MAG HS7, IKA Poland Sp. z o.o., Warsaw, Poland) at room temperature for 24 h until the chitosan was completely dissolved. The concentration of chitosan in the stock solution was 10 mg/mL (*w*/*v*).

#### 3.1.2. Phytopathogens

Eight fungal and two bacterial phytopathogens causing potato diseases were used in the research (Table 3).

The fungal strains were cultivated on potato dextrose agar (PDA) (Merck, Darmstadt, Germany). The bacterial strains were cultivated on tryptic soy agar (TSA) (Merck, Darmstadt, Germany). The strains were stored at 4 °C. Suspensions of the fungal strains were prepared by resuspending spores obtained from the pure culture on PDA in 0.85% NaCl with Tween (0.02%). The final concentrations of spores were standardized densitometrically (DEN1, Merck) and established as 10^6^ CFU/mL. Bacterial cell suspensions were prepared from the pure cultures on TSA and standardized densitometrically to a final concentration of 10^6^ CFU/mL.

#### 3.1.3. Potato Material

The experiments were performed using “Impresja” seed potatoes (*S. tuberosum* L.) acquired from Zamarte Potato Breeding Sp. z o. o. (IHAR Group, Kamieńsk Krajeński, Poland, 53°3505800 N, 17°2805400 E). “Impresja” is an early variant of potato, which is grown widely in Poland and has potential for cultivation in various countries.

### 3.2. In Vitro Evaluation of Antimicrobial Activities of Chitosan Solutions against Potato Pathogens

The Minimal Inhibitory Concentration (MIC) of each chitosan formulation was determined using the macro-broth dilution method. First, 1 mL of each chitosan solution was serially diluted in 1 mL of tryptic soy broth (TSB) (Merck, Darmstadt, Germany) for bacteria or malt extract broth (MEB) (Merck, Darmstadt, Germany) for moulds, to obtain concentrations of 5–0.00977 mg/mL. Then, 1 mL of phytopathogen suspension was added and the solutions were incubated for 1–3 days at 25 °C. The incubation time depended on the group of microorganisms used. The visible growth of the bacteria and moulds was evaluated densitometrically to determine the MIC values for each pathogen–chitosan combination. To determine the Minimal Bactericidal/Fungicidal Concentration (MBC/MFC), the cultures were inoculated from liquid samples (marked as MIC) onto TSA or MEA plates and incubated 1–5 days at 25 °C. The cidal concentration was established when no growth on the TSA (bacteria) or MEA (moulds) was noted. Three repetitions were performed or each pathogen–chitosan solution combination. Similarly, MIC was determined for control samples—lactic and succinic acid. These acids at the concentrations used to dissolve chitosan did not show any inhibitory effect on the growth of the tested phytopathogens. The most active formulation in terms of antibacterial and antifungal properties was selected for further studies. 

### 3.3. Cytotoxicity of Selected Chitosan Formulation against Selected Cell Lines

#### 3.3.1. Cell Culturing

The *S. frugiperda* insect normal larval ovaries cell line Sf-9, the normal immortalized human keratinocyte cell line HaCaT (original material created by Prof. Dr. Petra Boukamp and Dr. Norbert Fusenig) [70], and the human colon adenocarcinoma cell line Caco-2 were used to test the cytotoxicity of the selected chitosan solution. The Sf-9 cell line was purchased from Thermo Fisher Scientific (Waltham, MA, USA). The HaCaT and Caco-2 cell lines were purchased from Cell Line Service GmbH (Eppelheim, Germany).

HaCaT and Caco-2 cells were cultured in T75 flasks in DMEM (Sigma-Aldrich, St. Louis, MO, USA) supplemented with 10% FBS (foetal bovine serum) (Invitrogen Thermo Fisher Scientific, Waltham, MA, USA), 4 mM GlutaMAX^TM^ (Thermo Fisher Scientific, Waltham, MA, USA), 25 mM HEPES buffer (Sigma-Aldrich, St. Louis, MO, USA), and 100 µg/mL streptomycin/100 IU/mL penicillin mixture (Sigma-Aldrich, St. Louis, MO, USA). HaCaT and Caco-2 cells were cultured as a monolayer for 5–10 days at 37 °C in 5% CO_2_. The Sf-9 cells were cultured in Sf-900™ III Serum-Free Medium at 27 °C in a non-humidified, ambient-air-regulated incubator for 10–14 days. Every 2–3 days, the cells were washed with PBS buffer without calcium or magnesium (pH 7.2) (Sigma-Aldrich, St. Louis, MO, USA) and fresh medium was added. Confluent cells (Caco-2 and HaCaT) were detached with TrypLE^TM^ Express (6 min) or with gentle pipetting (Sf-9), centrifuged (182× *g*, 3–5 min), and decanted. The pellets were resuspended in fresh culture medium. Finally, the cells were counted using a haemocytometer and their viability was measured by trypan blue staining. For the results to be considered satisfactory, at least 80–90% viability was required.

#### 3.3.2. MTT Assay

The cytotoxicity of the MM-L chitosan solution was determined using the MTT (3-(4,5-dimethylthiazol-2-yl)-2,5-diphenyltetrazolium bromide) assay. First, 2 × 10^4^ (Sf-9) or 1 × 10^4^ (HaCaT and Caco-2) cells/well were placed in a culture medium in 96-well plates and incubated under appropriate conditions (above described) for 24 h. The medium was aspirated, and the following final concentrations of MM-L chitosan solution were added to the wells [mg/mL]: 0.016, 0.031, 0.063, 0.125, 0.25, 0.5, 1, and 2. Each concentration was tested in 4 replicates. The cell lines in an appropriate supplemented culture medium were used as a negative control. The cells were exposed to MM-L chitosan solution dilutions for 24 h in optimal conditions (28 °C for Sf-9, 37 °C for HaCaT and Caco-2). After this time, the samples were aspirated and MTT (0.5 mg/mL in PBS) (Sigma-Aldrich, St. Louis, MO, USA) was added to each well. The plates were incubated for another 3 h. Then, the MTT was aspirated and DMSO was added to dissolve the formazan precipitates. Absorbance was measured at 550 nm (using a 620 nm reference filter) in a microplate reader (TriStar² LB 942, Berthold Technologies GmbH & Co. KG, Bad Wildbad, Germany). The absorbance of the negative control represented 100% cell viability. Cell viability was calculated according to the equation.

Cell viability (%) = (S_OD_/C_OD_) × 100%,

where S is the optical density (OD) of the tested sample and C is an OD of the control sample. Cytotoxicity was calculated according to the equation

Cytotoxicity (%) = 100 − cell viability.

### 3.4. In Situ Antimicrobial Assay of Chitosan Solution against Potato Pathogens on Potatoes

We performed an in situ antimicrobial assay on the “Impresja” seed potatoes, according to a method we described in previous research [18,22,27]. Briefly, the potatoes were cut into 5 mm wide and 5 mm deep samples using a cork borer. Then, the samples were dipped in MM-L chitosan solution for 5 min and inoculated with 20 µL of each pathogen suspension, prepared according to the method described in Section 3.1.2. Control samples were only inoculated with the bacteria or mould suspension. The potato samples were incubated for 14 days at 25 °C. Inhibition of pathogen infestation was measured in comparison to the controls, according to the equation

Inhibition (%) = (C − T)/C × 100,

where C is the percentage area of pathogen infestation on the control potatoes and T is the percentage area of pathogen infestation on potatoes treated with MM-L chitosan solution and inoculated with the pathogen. The infested area was measured after cutting the seed potatoes in half. Each test was conducted in triplicate.

### 3.5. Greenhouse Experiments 

#### 3.5.1. Plant Cultivation Conditions

Suspensions of *A. tenuissima*, *F. sambucinum*, and *P. carotovorum* as mixtures (volume proportion 1:1:1) were used to inoculate the potato seeds after MM-L chitosan solution treatment. Following a 14-day incubation period, the potatoes were grown in a ventilated greenhouse maintained at 20 °C. They were placed in a microcosm measuring 30 cm in height, 40 cm in width, and 10 cm in depth, which was filled with 10 L of standard horticultural substrate mixes and a universal complete fertilizer containing macro- and microelements (YaraMila Complex; Yara) at a dose of 2 kg/m^3^. The walls of the microcosms were transparent, which allowed us to observe tuber germination and root growth. The potato plants were watered with tap water as required during the growing season [71].

#### 3.5.2. Growth and Physiological Activity of Plants

The potatoes planted in microcosms were checked every 2 days for the first 24 days, then every 6 days for the remaining period, to assess the germination, growth, and physiological activity of the plants. The following parameters were observed:

- Percentage of germinating tubers; kinetics of the plant growth, assessed by measuring the stem length;
- Quality and kinetics of root growth, assessed by measuring the lengths of the roots until they reached 30 cm;
- Kinetics of the root system growth, assessed by measuring the percentage of the profile area filled with roots;
- Intensity of gas exchange, including net photosynthesis, stomatal conductivity, transpiration, and intercellular CO_2_ concentration, examined throughout the period of most dynamic growth of potato plants in the highest most fully developed leaves, using a TPS-2 device (PP Systems, Amesbury, MA, USA);
- Index of chlorophyll content, examined throughout the period of most dynamic growth of potato plants in the highest most fully developed leaves, using a Minolta SPAD-502 device (Konica Minolta, Tokyo, Japan) [72,73,74].

### 3.6. Statistical Analysis

Statistical analysis was performed using Statistica 13.1 (Statsoft, Tulsa, OK, USA) [75]. All measurements of potato growth and physical activity were performed in triplicate, with four plants in each repetition. The results are reported as the mean ± standard deviation. Measurements were compared using a one-way ANOVA with a Duncan’s multiple range test at a significance level of *p* < 0.05. For other measurements, the results are presented as the mean ± standard deviation.

## 4. Conclusions

In this study, we investigated different formulations of chitosan as potential biocontrol agents against selected microbial diseases of potato plants. We also assessed the toxicity of chitosan against both human and insect cell lines. It was shown that antimicrobial formulations of chitosan significantly inhibited the growth of potato pathogens, but the final effect depended on the type of chitosan and the types of acidic solvent used. The results for selected pathogen monocultures are promising. Future work should evaluate the activity of chitosan against other potato pathogens and their mixed cultures. It is worth emphasizing that the tested chitosan formulations were non-toxic to human cells, but potential toxicity to insects was observed. In summary, this study opens the way to the development of new biopesticides for the effective protection of potato crops.

## Figures and Tables

**Table 1:** In vitro antimicrobial activity of chitosan solutions against potato phytopathogens.

Phytopathogens	MIC/MBC or MFC [mg/mL]					
	HM-L	MM-L	LM-L	HM-S	MM-S	LM-S
*Fusarium oxysporum*	0.1041/0.4167	0.1041/0.2083	0.05208/0.2083	0.1041/0.4167	0.1041/0.2083	0.1041/0.2083
*Fusarium sambucinum*	0.05208/0.4167	0.4167/0.8333	0.05208/0.2083	0.1041/0.4167	0.4167/1.666	0.4167/0.8333
*Alternaria alternata*	3.333/nd	0.4167/nd	3.333/nd	nd/nd	nd/nd	nd/nd
*Alternaria solani*	0.05208/nd	0.1041/0.4167	0.1041/0.4167	0.1041/0.4167	0.1041/0.8333	0.2083/1.666
*Alternaria tenuissima*	3.333/nd	0.1041/nd	0.8333/1.666	nd/nd	0.4167/3.333	0.2083/1.666
*Colletotrichum coccodes*	nd/nd	nd/nd	nd/nd	nd/nd	nd/nd	nd/nd
*Rhizoctonia solani*	nd/nd	1.666/3.333	1.666/nd	nd/nd	3.333/nd	nd/nd
*Phoma exigua*	0.05208/0.2083	0.1041/0.1041	0.1041/0.1041	0.2083/0.2083	0.1041/0.2083	0.1041/0.4167
*Pectobacterium carotovorum*	0.4167/3.333	0.2083/nd	1.666/nd	0.4167/3.333	0.2083/1.666	0.4167/3.333
*Streptomyces scabiei*	3.333/nd	0.8333/3.333	3.333/nd	3.333/3.333	3.333/nd	3.333/nd

MIC—Minimal Inhibitory Concentration; MBC/MFC—Minimal Bactericidal Concentration/Minimal Fungicidal Concentration; HM-L—High molecular weight chitosan dissolved in lactic acid; MM-L—Medium molecular weight chitosan dissolved in lactic acid; LM-L—Low molecular weight chitosan dissolved in lactic acid; HM-S—High molecular weight chitosan dissolved in succinic acid; MM-S—Medium molecular weight chitosan dissolved in succinic acid; LM-S—Low molecular weight chitosan dissolved in succinic acid; nd—not detected (the highest concentration of chitosan solution did not inhibit the growth of phytopathogen).

**Table 2:** Inhibition effects of MM-L chitosan solution on potato phytopathogens. A1—potato infected with *F. oxysporum*; B1—potato treated with MM-L chitosan solution and infected with *F. oxysporum*; A2—potato infected with *A. tenuissima*; B2—potato infected treated with MM-L chitosan solution and infected with *A. tenuissima*.

**Phytopathogens**	**Inhibition of Potato Infestation (%)**	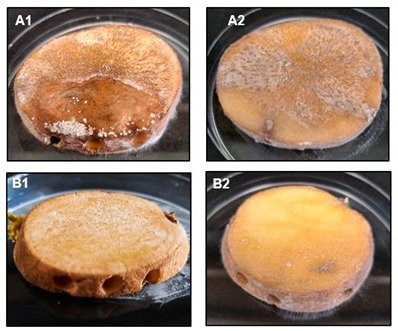
*Fusarium oxysporum*	100 ± 0	
*Fusarium sambucinum*	40 ± 5	
*Alternaria alternata*	20 ± 0	
*Alternaria solani*	55 ± 10	
*Alternaria tenuissima*	50 ± 5	
*Colletotrichum coccodes*	0 ± 0	
*Rhizoctonia solani*	80 ± 5	
*Phoma exigua*	80 ± 10	
*Pectobacterium carotovorum*	25 ± 0	
*Streptomyces scabiei*	40 ± 0	

**Table 3:** Phytopathogens used in the study.

Fungi	**Strain**	**Origin**
	*Fusarium sambucinum* DSM 62397 *Alternaria tenuissima* DSM 63360 *Phoma exigua* DSM 62040 *Colletotrichum coccodes* DSM 62126 *Rhizoctonia solani* DSM 22843	German Collection of Microorganisms and Cell Cultures GmbH (DSMZ, Braunschweig, Germany)
	*Alternaria alternata* ŁOCK 408	Collection of Pure Cultures of Industrial Microorganisms ŁOCK at Lodz University of Technology (Łódź, Poland)
	*Fusarium oxysporum* Z 154 *Alternaria solani* Z 184	Plant Breeding and Acclimatization Institute (IHAR)—National Research Institute (Radzików, Poland)
Bacteria	*Streptomyces scabiei* DSM 40778	German Collection of Microorganisms and Cell Cultures GmbH (DSMZ, Braunschweig, Germany)
	*Pectobacterium carotovorum* PCM 2056	Polish Collection of Microorganisms at the Hirszfeld Institute of Immunology and Experimental Therapy of the Polish Academy of Sciences (Wrocław, Poland)

## Data Availability

Data are contained within the article.
